# Circulating autoantibodies against neuroblastoma suppressor of tumorigenicity 1 (NBL1): A potential biomarker for coronary artery disease in patients with obstructive sleep apnea

**DOI:** 10.1371/journal.pone.0195015

**Published:** 2018-03-29

**Authors:** Takuma Matsumura, Jiro Terada, Taku Kinoshita, Yoriko Sakurai, Misuzu Yahaba, Kenji Tsushima, Seiichiro Sakao, Kengo Nagashima, Toshinori Ozaki, Yoshio Kobayashi, Takaki Hiwasa, Koichiro Tatsumi

**Affiliations:** 1 Department of Respirology, Graduate School of Medicine, Chiba University, Chiba, Chiba, Japan; 2 Department of Global Clinical Research, Graduate School of Medicine, Chiba University, Chiba, Chiba, Japan; 3 Laboratory of DNA Damage Signaling, Chiba Cancer Center Research Institute, Chiba, Chiba, Japan; 4 Department of Cardiovascular Medicine, Graduate School of Medicine, Chiba University, Chiba, Chiba, Japan; 5 Department of Biochemistry and Genetics, Graduate School of Medicine, Chiba University, Chiba, Chiba, Japan; Osaka University Graduate School of Medicine, JAPAN

## Abstract

**Objective:**

Although severe obstructive sleep apnea (OSA) is an important risk factor for atherosclerosis-related diseases including coronary artery disease (CAD), there is no reliable biomarker of CAD risks in patients with OSA. This study aimed to test our hypothesis that circulating autoantibodies against neuroblastoma suppressor of tumorigenicity 1 (NBL1-Abs) are associated with the prevalence of CAD in patients with OSA.

**Methods:**

Eighty-two adults diagnosed with OSA by polysomnography, 96 patients with a diagnosis of acute coronary syndrome (ACS) and 64 healthy volunteers (HVs) were consecutively enrolled. Serum samples were collected from patients with OSA at diagnostic polysomnography and from patients with ACS at disease onset. Serum NBL1-Ab level was measured by amplified luminescence proximity homogeneous assay and its association with clinical variables related to atherosclerosis was evaluated.

**Results:**

NBL1-Ab level was significantly elevated in patients with both OSA and ACS compared with HVs. Subgroup analyses showed that NBL1-Ab level was markedly higher in patients with severe OSA and OSA patients with a history of CAD. Weak associations were observed between NBL1-Ab level and apnea-hypopnea index, age, mean SpO_2_ and arousal index, whereas significantly higher NBL1-Ab levels were observed in OSA patients with a history of CAD than in those without a history of CAD. Sensitivity analysis using a logistic regression model also demonstrated that increased NBL1-Ab levels were associated with the previous history of CAD in patients with OSA.

**Conclusions:**

Elevated NBL1-Ab levels may be associated with the prevalence of CAD in patients with OSA, which needs to be confirmed further.

## Introduction

Patients with obstructive sleep apnea (OSA) experience repetitive upper airway obstruction during sleep, which causes frequent sleep fragmentation and nocturnal intermittent hypoxia (IH). Sleep fragmentation and IH promote oxidative stress by accelerating the production of reactive oxygen species. They also enhance inflammation of systemic, vascular and adipose tissue by elevating levels of inflammatory cytokines and cause upregulation of blood pressure with increased sympathetic activity [1, 2]. These OSA-related reactions induce endothelial dysfunction, leading to the development of atherosclerosis and eventually to coronary artery disease (CAD), strokes and atherosclerosis-related deaths, especially in patients with severe OSA [3–5].

OSA treatment with continuous positive airway pressure (CPAP) therapy can reduce the impact of inflammation, blood lipid values and elevated blood pressure in patients with OSA [6–8]. Despite its benefits, adherence to CPAP therapy has been reported to range from 29% to 85%, which is not ideal [9]. Therefore, biomarkers of patients with OSA who require more careful and intensive treatment (i.e. greater adherence to CPAP therapy) are needed. Several markers of inflammation and endothelial dysfunction have been proposed as candidate markers for OSA patients with atherosclerosis-related diseases (e.g. soluble tumour necrosis factor receptor, tumour necrosis factor-beta, interleukin-6 and soluble intercellular cell adhesion molecule-1). However, there is no consensus on the optimal biomarker of CAD risks in OSA. In clinical practice, although the ankle-brachial pressure index, brachial-ankle pulse wave velocity (baPWV), cardio-ankle vascular index, carotid intima-media thickness (IMT) and funduscopic examination have been used to evaluate arterial atherosclerosis, predicting the development of fatal acute coronary syndrome (ACS) is difficult.

Recently, circulating (i.e. serum) autoantibodies against atherosclerosis-specific antigens have been considered as novel biomarkers for evaluating atherosclerosis-related diseases. We have previously described the presence of the autoantibodies recognized by IgG antibodies in the sera of patients with atherosclerosis-related diseases such as myocardial infarction, stroke and diabetes [10, 11]. Among them, we have reported circulating autoantibodies against coatomer protein complex subunit epsilon, which is a potential biomarker of cardiovascular and cerebrovascular risk in patients with OSA [12]. Additionally, we have focused on autoantibodies against members of the bone morphogenetic protein (BMP) family because BMP may be associated with atherosclerosis and cardiovascular disease (CVD) [13]. BMP signalling is activated by BMP1, which degrades BMP antagonist chordin [14], resulting in atherosclerosis. The serum level of autoantibodies against BMP1 has been reported as a potential marker of CVD, ischemic stroke and transient ischemic attack [10, 15]. Other studies suggest that BMP2 and BMP4 are linked to the development of atherosclerosis [13], and BMP antagonist neuroblastoma suppressor of tumorigenicity 1 (NBL1, accession number X66872.1) specifically antagonizes BMP2, BMP4 and BMP14 [16, 17]. Thus, it is conceivable that there exists a functional relationship between NBL1 and atherosclerosis.

In this study, we evaluated serum autoantibodies against NBL1 (NBL1-Ab) level in patients with OSA and ACS, and tested the hypothesis that NBL1-Abs are increased in OSA patients with a history of CAD.

## Material and methods

### Ethical approval

All study procedures involving human participants were approved by the ethical standards of the Ethical Review Board of the Chiba University, Graduate School of Medicine (approval number 973). The study conformed to the ethical principles of 1964 Helsinki declaration and subsequent amendments. All study participants provided written informed consent.

### Study subjects

Eighty-two Japanese adults, 56 men and 26 women with a median age of 59.0 years, who were diagnosed with OSA by polysomnography (PSG) in our hospital between June 2012 and January 2014, were studied. Ninety-six patients with ACS, 81 men and 15 women, with a median age of 67.0 years, who were diagnosed in our hospital, were recruited at the disease onset between April 2011 and March 2014. ACS was defined as acute myocardial infarction (AMI) or unstable angina pectoris (UAP). Sixty-four healthy volunteers (HVs), 38 men and 26 women, with a median age of 42.5 years and no history of CAD or OSA were control subjects. The history of CAD was defined as a previous clinical history of myocardial infarction or angina pectoris. All HVs were enrolled at Chiba University. OSA and ACS patients with autoimmune diseases and ACS patients with a history of sleep-related breathing disorders according to the International Classification of Sleep Disorders-Third Edition criteria were also excluded.

### Clinical assessment

Atherosclerosis risk factors, including age, sex, body mass index (BMI), smoking status, hypertension, diabetes, hyperlipidemia, CAD and stroke, were collected from patient clinical records. Patients were divided into three groups by smoking status (i.e. never smoked, ex-smoker and current smoker). Hypertension was defined as a history of systolic blood pressure > 140 mmHg, diastolic blood pressure > 90 mmHg or use of antihypertensive agents, and diabetes was defined as the use of antidiabetes therapy or a history of diabetes. Hyperlipidemia was defined as a history of total cholesterol > 220 mg/dL, triglycerides > 150 mg/dL or use of lipid-lowering agents. Stroke was defined as a history of cerebral infarction or cerebral haemorrhage.

PSG was scored according to the 2007 American Academy of Sleep Medicine alternative criteria. Apnea was defined as a reduction of nasal airflow to < 10% of the baseline for ≥ 10 s. Hypopnea was defined as a reduction in the nasal airflow signal amplitude of ≥ 50% for ≥10 s in association with either a ≥ 3% oxygen desaturation or electroencephalographic arousal. OSA was defined as an apnea-hypopnea index (AHI) of five or more events per hour combined with predominantly obstructive respiratory events. OSA severity was classified according to AHI values as mild, 5–15; moderate, > 15–30 or severe, > 30.

### Blood sampling/purification and analysis

Blood samples were collected from patients with OSA on PSG evaluation and from HVs during their medical checkup. Blood was collected from patients with ACS at the diagnosis of AMI or UAP during emergency admission for percutaneous coronary intervention or coronary artery bypass grafting. Sera were centrifuged at 3000 g for 10 min at room temperature and the supernatant was stored at −80°C until use. Repeated thawing and freezing of the samples was avoided.

A full-length *NBL1* cDNA was used to construct the expression vector for glutathione-S-transferase (GST)-tagged NBL1 protein. The cDNA sequence between positions 267 and 731 was inserted into *Eco* RI/*Xho* I site of pGEX-2T (GE Healthcare Life Sciences, Pittsburgh, PA, USA), which produces the truncated NBL1 protein (from amino acid residue 25 to 178) lacking its potential signal peptide sequence. GST-NBL1 protein was purified as described previously [10, 15].

Amplified luminescence proximity homogeneous assay (AlphaLISA) was performed in 384-well microtiter plates (white opaque ProxiPlate^™^, PerkinElmer, Waltham, MA, USA) containing 2.5 μL of 1/100-diluted sera and 2.5 μL of GST or a GST-fusion protein (10 μg/mL) in AlphaLISA buffer (25 mM HEPES, pH 7.4, 0.1% casein, 0.5% Triton X-100, 1 mg/mL dextran-500 and 0.05% Proclin-300). The resulting reaction mixture was incubated at room temperature for 6–8 h. Anti-human IgG-conjugated acceptor beads (2.5 μL of 40 μg/mL) and glutathione-conjugated donor beads (2.5 μL of 40 μg/mL) were added, and the samples were incubated for an additional 14 days at room temperature in the dark. Chemiluminescence was read on an EnSpire Alpha microplate reader (PerkinElmer) as previously described [11, 15]. Specific reactions were calculated by subtracting the Alpha values of GST control samples from those of samples containing GST-fusion proteins.

### Statistical analysis

For the baseline variables, summary statistics were constructed using medians and interquartile ranges for numerical data and frequencies, respectively, and proportions for categorical data. Patient characteristics were compared using the Fisher’s exact test for categorical data and the Mann—Whitney U or Kruskal—Wallis test for numerical data, as appropriate. NBL1-Ab levels in patients with OSA, patients with ACS and HVs were compared using the Kruskal—Wallis test. A post hoc analysis was performed for all pairwise comparisons using the Steel—Dwass method. Subgroup analyses were performed for NBL1-Ab levels by OSA severity (mild, moderate and severe) and by the presence or absence of CAD history. The subgroup analyses were conducted in the same manner as the pooled analysis. Correlations were evaluated using the Spearman’s correlation analysis, and the Fisher’s exact test was used to determine the significance of differences in the proportions of groups. The cut-off value of NBL1-Ab level for the prevalence of CAD among all patients with OSA was determined to maximize the sum of sensitivity and specificity by receiver operating characteristic (ROC) curve analysis. Univariate and multivariate logistic regression analyses were used to identify the set of variables that could classify patients according to CAD status. Nine covariates were included in the models, including age (years), obesity (BMI ≥ 25 kg/m^2^), smoking (current or ex-smoker), hypertension, diabetes, hyperlipidemia, stroke, severe OSA (AHI > 30) and elevated NBL1-Ab. All tests were two-tailed, and statistical significance was defined as a p value < 0.05. All statistical analyses were performed using JMP Pro 12.2.0 software (SAS Institute Inc., Cary, NC, USA). (Whole raw data sheet is provided in S1 Table)

## Results

### Patient characteristics

**Fig 1:**
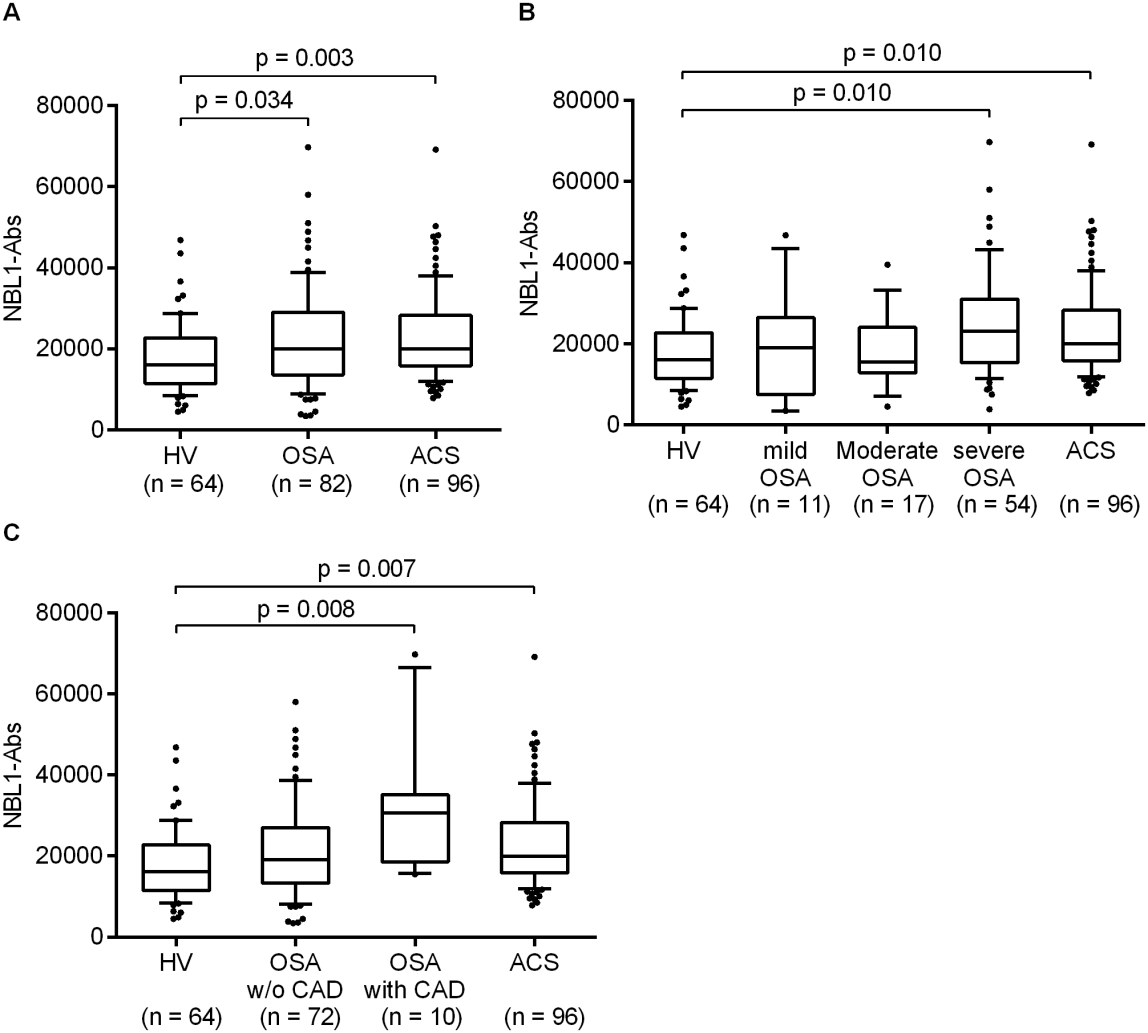
Association between NBL1-Ab level and disease. Pooled OSA group data were compared with those of patients with ACS and HVs. Significant differences were observed among the three groups using the Kruskal—Wallis test (p < 0.004). Post hoc analysis using the Steel—Dwass test revealed significant differences between both patients with OSA and ACS and the HVs (Fig 1A). Patients with OSA were stratified by OSA severity (Fig 1B) or by having a history of CAD (Fig 1C) and then compared with patients with ACS and HVs. Significant differences were observed in both comparisons using the Kruskal—Wallis test (p < 0.003 and p < 0.001). Post hoc analysis using the Steel—Dwass test revealed significant differences between both patients with severe OSA and OSA patients with CAD versus HVs. Horizontal lines represent medians, boxes represent the 25th and 75th percentiles, whiskers represent the 10th and 90th percentiles and dots represent outliers. ACS, acute coronary syndrome; CAD, coronary artery disease; HV, healthy volunteer; NBL1-Abs, autoantibodies against NBL1; OSA, obstructive sleep apnea; w/o, without.

Clinical characteristics of patients with OSA, patients with ACS and HVs are shown in Table 1. Sixty-four HVs, 82 patients with OSA (11 mild, 17 moderate and 54 severe OSA) and 96 patients with ACS (72 AMI and 24 UAP) were enrolled in this study. As compared to HVs, patients with OSA were significantly older and had a higher BMI index. The patients with ACS were significantly older and included more male subjects than HVs. A history of atherosclerosis-related diseases (e.g. hypertension or CAD) was more frequently observed in patients with OSA and ACS than in HVs. As shown in Fig 1A, a significantly higher serum NBL1-Ab level was observed in patients with OSA (p = 0.034) and patients with ACS (p = 0.003) than in HVs. There were no significant differences in the NBL1-Ab levels in patients with OSA and ACS (p = 0.864). Subgroup analysis found that only patients with severe OSA and a history of CAD had significantly higher NBL1-Ab levels than HVs had (p = 0.010, Fig 1B and p = 0.008, Fig 1C). In patients with mild-to-moderate severity OSA (Fig 1B) and those with no history of CAD (Fig 1C), the NBL1-Ab level was similar to that in HVs.

**Table 1:** Baseline characteristics of subjects.

	HV (n = 64)	OSA (n = 82)	ACS (n = 96)
Age (years)	42.5 (35.3–55.8)	59.0 (49.8–66.5)***	67.0 (60.0–73.0)***
Male sex	38 (59.4%)	56 (68.3%)	81 (84.4%)***
BMI (kg/m^2^)	23.1 (20.6–25.5)	25.9 (23.9–29.4)***	23.4 (21.3–25.2)
OSA severity			
Mild		11 (13.4%)	
Moderate		17 (20.7%)	
Severe		54 (65.9%)	
AHI (events/h)		36.7 (22.6–50.4)	
Mean SpO_2_ (%)		94.0 (93.0–96.0)	
Lowest SpO_2_ (%)		78.0 (69.0–83.0)	
Arousal index (events/h)		37.3 (22.2–50.3)	
Smoking status	(n = 60)		
Never smoked	42 (70.0%)	42 (51.2%)	30 (31.3%)
Ex-smoker	10 (16.7%)	34 (41.5%)	38 (39.6%)
Current smoker	8 (13.3%)	6 (7.3%)**	28 (29.2%)***
Hypertension	8 (12.5%)	30 (36.6%)**	45 (46.9%)***
Diabetes	1 (1.6%)	17 (20.7%)**	17 (17.7%)**
Hyperlipidemia	2 (3.1%)	22 (26.8%)*	14 (14.6%)*
Previous history of CAD	0 (0.0%)	10 (12.2%)**	14 (14.6%)***
Stroke	0 (0.0%)	5 (6.1%)	5 (5.2%)

Data are medians (interquartile range) for numerical data and n (%) for categorical data.

ACS, acute coronary syndrome; AHI, apnea-hypopnea index; BMI, body mass index; CAD, coronary artery disease; HV, healthy volunteer; OSA, obstructive sleep apnea.

*p < 0.05 versus HV,

**p < 0.01 versus HV,

***p < 0.001 versus HV.

### Association of NBL1-Ab level with clinical parameters of patients with OSA

**Fig 2:**
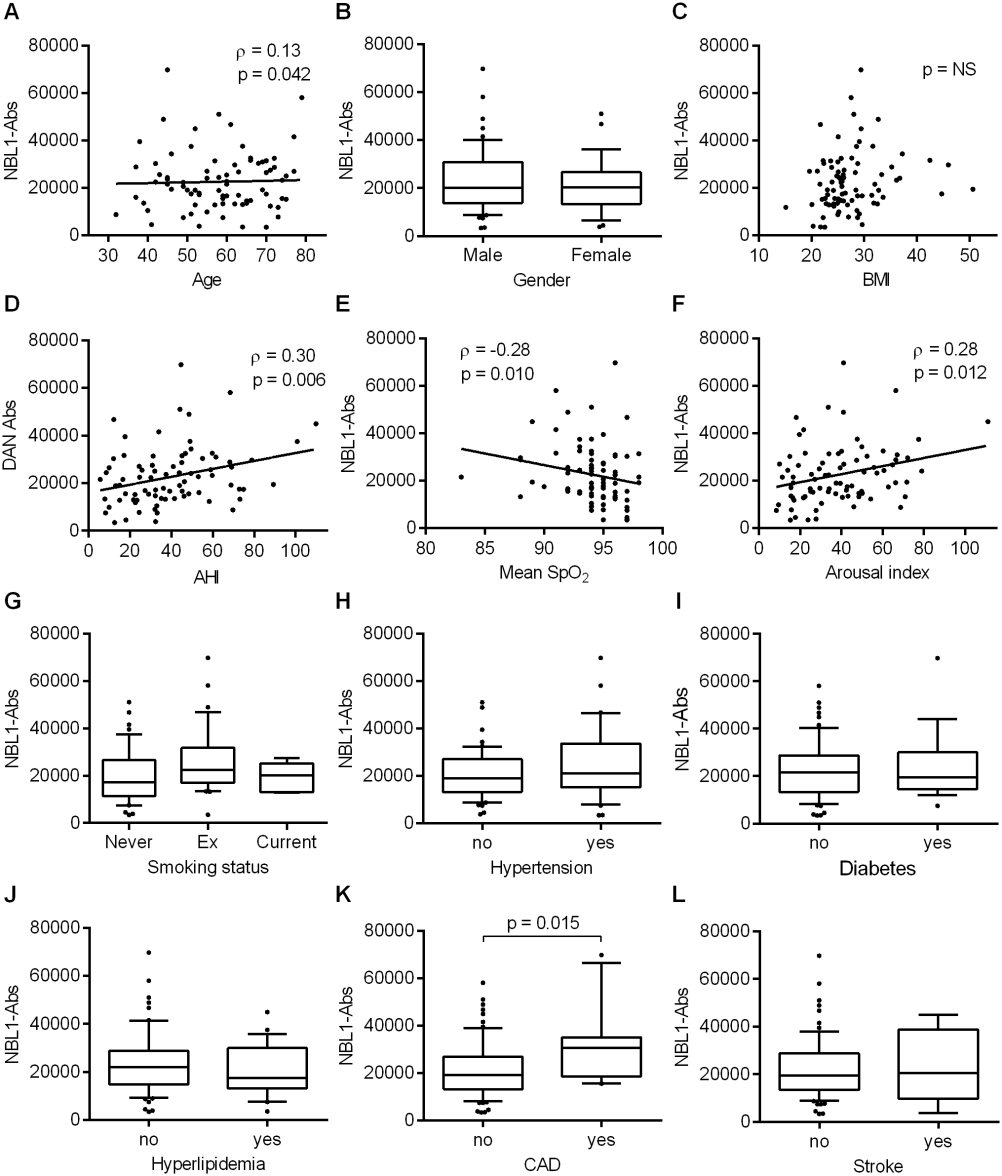
Association of NBL1-Ab with clinical parameters in patients with OSA. Correlations between NBL1-Abs and age (A), gender (B), BMI (C), AHI (D), mean SpO_2_ (E), arousal index (F), smoking status (G), hypertension (H), diabetes (I), hyperlipidemia (J), CAD (K), and stroke (L) were evaluated. Weak associations were observed between NBL1-Ab levels and age, AHI, mean SpO_2_ and arousal index. OSA patients with CAD had significantly higher NBL1-Ab levels than OSA patients without CAD. Spearman’s correlation analysis (A, C—F), Mann—Whitney U test (B, H-L) and Kruskal—Wallis test (G) were used. Horizontal lines represent medians, boxes represent the 25th and 75th percentiles, whiskers represent the 10th and 90th percentiles and dots represent outliers. AHI, apnea-hypopnea index; BMI, body mass index; CAD, coronary artery disease; NBL1-Abs, autoantibodies against NBL1; OSA, obstructive sleep apnea.

The relationships of NBL1-Ab level and clinical parameters in patients with OSA are shown in Fig 2. An association was observed between NBL1-Ab levels and AHI (ρ = 0.30, p = 0.006, Fig 2D), indicating a weak but significant dose response for NBL1-Ab levels in OSA. In addition, a very weak to weak association was observed between NBL1-Ab level and age (ρ = 0.13, p = 0.042, Fig 2A), mean SpO_2_ (ρ = −0.28, p = 0.010, Fig 2E) and arousal index (ρ = 0.28, p = 0.012, Fig 2F), whereas a significantly higher NBL1-Ab level was observed in patients with CAD than in those without CAD (p = 0.015, Fig 2K). No significant associations were found between NBL1-Ab level and sex, BMI, smoking status, hypertension, diabetes, hyperlipidemia or stroke. However, regarding absolute values of serum lipid levels, a weak association was observed between NBL1-Ab level and low-density lipoprotein (LDL) (ρ = 0.24, p = 0.034) and total cholesterol (ρ = 0.28, p = 0.014), whereas no significant associations were found between NBL1-Ab level and high-density lipoprotein and triglyceride level (data not shown).

**Fig 3:**
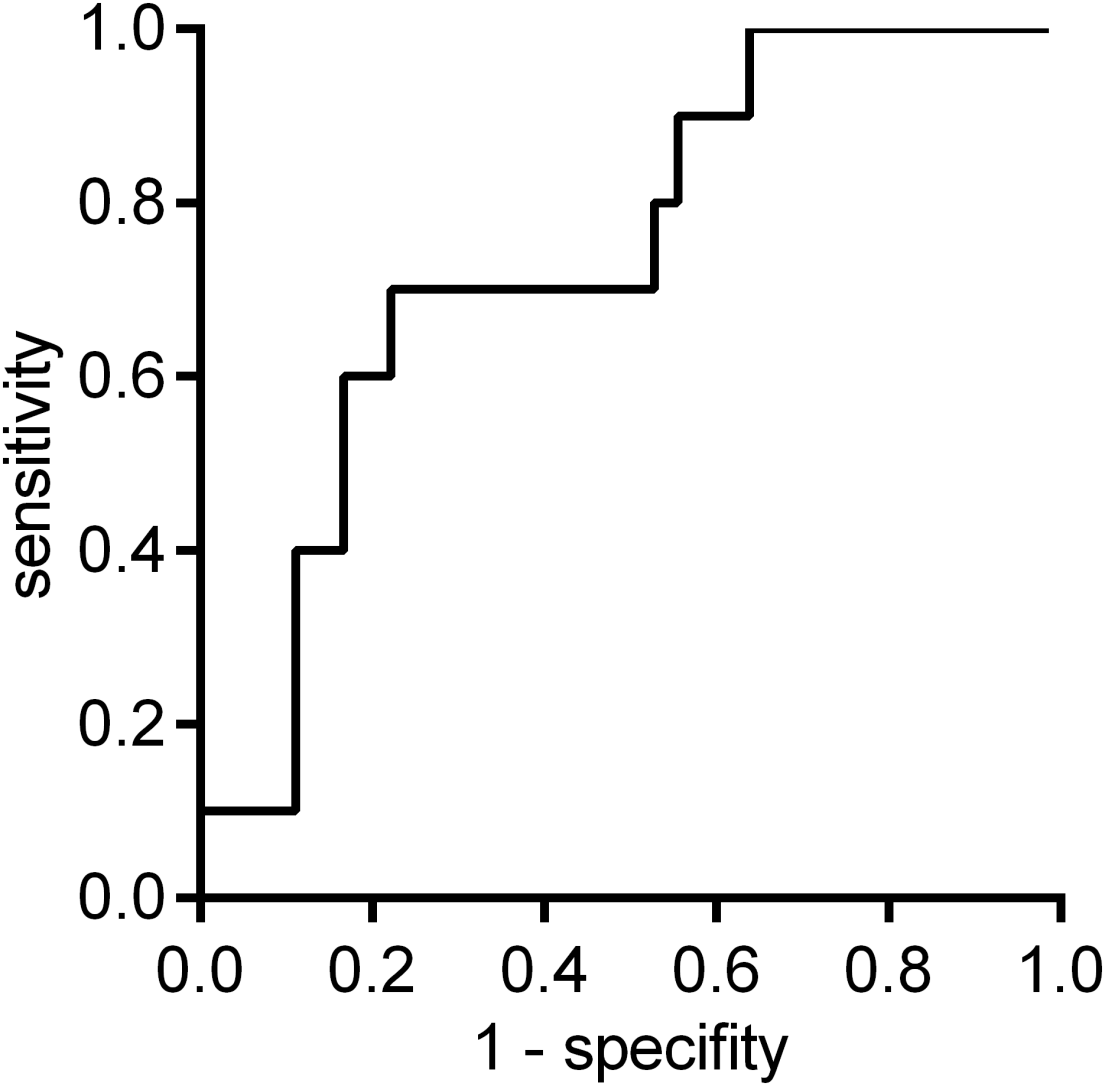
ROC curve analysis for the predictive value of NBL1-Abs for CAD among all OSA patients. The area under the curve was 0.739 (95% CI: 0.560–0.863). When the NBL1-Ab cut-off was 27512, the sensitivity of NBL1-Abs for predicting CAD was 70.0% and the specificity was 77.8% (n = 82; no. of events = 10). CAD, coronary artery disease; CI, confidence interval; NBL1-Abs, autoantibodies against NBL1; OSA, obstructive sleep apnea; ROC, receiver operating characteristic.

We performed a sensitivity analysis to identify the strength of the association of clinical parameters including NBL1-Ab level with the prevalence of CAD using univariate and multivariate logistic regression models as shown in Table 2. For patients with OSA, an optimal cut-off value of NBL1-Ab for predicting the prevalence of CAD history was determined to be 27512 by ROC curve analysis, with a sensitivity of 70.0% and a specificity of 77.8% (Fig 3). The area under the curve was 0.739 (95% confidence interval [CI]: 0.560–0.863). Using the NBL1-Abs cut-off value, univariate logistic regression revealed an association of elevated NBL1-Ab level with the prevalence of CAD (odds ratio [OR]: 8.17, 95% CI: 2.03–41.4, p = 0.003). Multivariate logistic regression analysis, which included variables with p < 0.10 in the univariate analysis (severe OSA and elevated NBL1-Ab levels), demonstrated an independent association of only elevated NBL1-Ab level with CAD (OR: 6.75, 95% CI: 1.63–34.9, p = 0.008). We also performed additional multivariate logistic regression analysis with three covariates; two same covariates in the multivariate analysis above and age, which was significantly associated with NBL1-Ab levels in Fig 2. The result did not change, and only elevated NBL1-Ab level was found to be associated with CAD (data not shown).

**Table 2:** Logistic regression of the prevalence of CAD in patients with OSA (n = 82; no. of events = 10).

	Univariate Analysis			Multivariate Analysis		
	OR	95% CI	p value	OR	95% CI	p value
Age (per year)	1.04	0.97–1.11	0.26			
Obesity (BMI ≥ 25, kg/m^2^)	0.80	0.21–3.36	0.75			
Smoking (Current or Ex-smoker)	2.76	0.71–13.6	0.15			
Hypertension	0.71	0.14–2.81	0.64			
Diabetes	3.03	0.69–12.2	0.13			
Hyperlipidemia	1.20	0.24–4.79	0.81			
Stroke	5.75	0.68–40.2	0.10			
Severe OSA (AHI > 30)	5.40	0.94–102.3	0.06	3.63	0.56–71.1	0.19
Elevated NBL1-Ab levels (≥ 27512)^†^	8.17	2.03–41.4	0.003	6.75	1.63–34.9	0.008

^†^NBL1-Ab cut-off was 27512 based on ROC curve analysis.

AHI, apnea-hypopnea index; BMI, body mass index; CAD, coronary artery disease; CI, confidence interval; NBL1-Abs, autoantibodies against NBL1; OSA, obstructive sleep apnea; OR, odds ratio; ROC, receiver operating characteristic.

## Discussion

The major finding of this study was that NBL1-Ab level was significantly elevated in patients with both OSA and ACS compared with HVs. Subgroup analyses showed that NBL1-Ab level was significantly higher in patients with severe OSA and OSA patients with a past history of CAD. Weak associations were observed between NBL1-Ab level and age, AHI, mean SpO_2_ and arousal index, whereas a significantly increased NBL1-Ab level was observed in OSA patients with CAD compared with those without CAD.

Regarding circulating autoantibodies for patients with atherosclerosis-related diseases, some antibodies have been previously reported as markers, including autoantibodies against phospholipids in patients with ACS [18], apolipoprotein A-1 in patients with increased atherosclerotic plaque vulnerability [19] and oxidized LDL (ox-LDL), which is associated with plaque formation and coronary risk, in some patients with systemic lupus erythematosus [20]. We have previously identified several atherosclerosis antigens serologically using recombinant cDNA expression cloning, and found a close association of the levels of antibodies against several autoantigens (e.g. RPA2, ATP2B4 and BMP1) with atherosclerosis-related diseases and risk factors of atherosclerosis-related diseases such as stroke, hypertension and smoking habits [10, 15]. Based on these observations, we hypothesized that certain antibody markers have association with past CAD in patients with OSA, which was confirmed in this present study.

*NBL1*, a member of the family of differential screening-selected gene aberrative in neuroblastoma *(DAN)* [21], is associated with not only neuroblastoma but also with remodelling of vessels by inhibiting pulmonary arterial smooth muscle cell proliferation [22]. One of the main roles of DAN family members including NBL1 is to act as specific antagonists of BMPs. BMPs have initially been found to induce bone formation and later identified as a multifunctional cytokine associated with anaemia, progressive bone formation and various cancers [23]. Among BMP family members, BMP2 and BMP4 are mediators of endothelial inflammation in response to shear stress, oxidative stress and pro-inflammatory cytokines [24, 25]. Clinically, serum BMP2 has been correlated with plaque burden and coronary artery calcification in patients with type 2 diabetes [26]. BMP4 has been shown to stimulate vascular endothelial growth factor synthesis [27]. Infusion of BMP4 induced vascular nicotinamide adenine dinucleotide phosphate oxidases and impaired vasodilation, leading to hypertension [28]. As NBL1 is a specific antagonist of BMP2 and BMP4, both of which promote atherosclerosis, NBL1 is capable to inhibit atherosclerosis. As NBL1 would be elevated concomitantly with BMP in atherosclerotic lesions, its autoantibodies (NBL1-Abs) may increase subsequently. Indeed, ox-LDL stimulation induced an increase in BMP2 in aortic valve interstitial cells, which corresponds with the association of LDL and NBL1-Abs in this study [29]. Therefore, NBL1-Ab may have the potential to be an optional marker of atherosclerosis in addition to other conventional investigations. However, a prospective study of patient groups without overlap is required to confirm the utility of NBL1-Ab, because ACS patients with OSA were excluded only by patient histories in this study, and thus, some patients with ACS may have had undiagnosed OSA.

Considering the significance of elevated NBL1-Abs in this study, a comparison between NBL1-Abs in OSA and ACS should be performed. Similar to all patients with ACS, patients with AMI without a past history of CAD (i.e. first episode of AMI) also had higher NBL1-Ab levels than HVs (p = 0.002). Because it generally takes several weeks for the production of IgG antibodies in response to antigen exposure and because sera were collected from patients when AMI was diagnosed, NBL1-Abs might be produced before the onset of AMI. Additionally, NBL1-Ab levels were significantly higher in OSA patients with a history of CAD than in those without a history of CAD. Accordingly, it appears that NBL1-Abs might have potential to be used as a biomarker of a past history of CAD and also as a predictive biomarker of CAD in patients with OSA. Among 72 OSA patients without a history of CAD, 16 patients in the present study showed elevated NBL1-Ab levels (>cut-off value, data are not shown). From these results, it is indicated that the 16 patients may require more clinical attention (e.g. increased adherence to CPAP therapy). Regarding the practical utility for evaluation of atherosclerosis in patients with OSA, coronary artery calcification, carotid IMT, baPWV and flow-mediated dilation are often used [30, 31]. However, the autoantibody assay described in this study is non-invasive and more convenient (only requires a serum sample) than the existing tests. Therefore, monitoring circulating NBL1-Ab levels might be useful for evaluating the risk of lethal events in patients with OSA; however, prospective cohort studies are needed to confirm its effectiveness as a marker for atherosclerosis risk.

This study has several limitations. First, ACS patients with sleep-related breathing disorders were excluded only by evidence obtained from patient histories not by PSG or home sleep tests. Similarly, the history of OSA in HVs were excluded by asking if they had any kind of diseases or medical examination history and not by conducting specific tests (e.g. PSG or home sleep tests) or by questioning clinical symptoms of OSA (e.g. snoring, daytime sleepiness). Consequently, some patients with ACS and HVs may have had sleep-related breathing disorders. Second, the number of OSA patients with CAD was relatively small. It is possible that the NBL1-Ab cut-off for the prevalence of CAD in all patients with OSA was inaccurate. AUC for predicting the prevalence of CAD history was 0.738, indicating that the ROC curve analysis had only fair utility. In addition, all patients with CAD were male. Therefore, we could not include gender as a covariate in the logistic regression analysis. Third, we did not evaluate atherosclerosis by conventional physiological tests. Finally, the analyses were not adjusted for potential confounding factors between patients and control HVs (e.g. age, BMI and history of atherosclerosis-related diseases).

In conclusion, patients with OSA had higher circulating NBL1-Ab levels than HVs, as was the case for patients with ACS. Serum NBL1-Ab levels were significantly increased in OSA patients with a history of CAD and severe disease. Further study is warranted to confirm whether elevated NBL1-Ab levels indicate increased risk of future CAD in patients with OSA.

## Supporting information

S1 Table Whole raw data sheet. (XLSX)
